# High-specificity detection of rare alleles with Paired-End Low Error Sequencing (PELE-Seq)

**DOI:** 10.1186/s12864-016-2669-3

**Published:** 2016-06-14

**Authors:** Jessica L. Preston, Ariel E. Royall, Melissa A. Randel, Kristin L. Sikkink, Patrick C. Phillips, Eric A. Johnson

**Affiliations:** Institute of Molecular Biology, University of Oregon, Eugene, Oregon USA; Institute of Ecology and Evolution, University of Oregon, Eugene, Oregon USA

**Keywords:** De novo mutations, Genetic heterogeneity, Laboratory adaptation, Minor alleles, Next-generation sequencing, PELE analysis, SNPs

## Abstract

**Background:**

Polymorphic loci exist throughout the genomes of a population and provide the raw genetic material needed for a species to adapt to changes in the environment. The minor allele frequencies of rare Single Nucleotide Polymorphisms (SNPs) within a population have been difficult to track with Next-Generation Sequencing (NGS), due to the high error rate of standard methods such as Illumina sequencing.

**Results:**

We have developed a wet-lab protocol and variant-calling method that identifies both sequencing and PCR errors, called Paired-End Low Error Sequencing (PELE-Seq). To test the specificity and sensitivity of the PELE-Seq method, we sequenced control *E. coli* DNA libraries containing known rare alleles present at frequencies ranging from 0.2–0.4 % of the total reads. PELE-Seq had higher specificity and sensitivity than standard libraries. We then used PELE-Seq to characterize rare alleles in a *Caenorhabditis remanei* nematode worm population before and after laboratory adaptation, and found that minor and rare alleles can undergo large changes in frequency during lab-adaptation.

**Conclusion:**

We have developed a method of rare allele detection that mitigates both sequencing and PCR errors, called PELE-Seq. PELE-Seq was evaluated using control *E. coli* populations and was then used to compare a wild *C. remanei* population to a lab-adapted population. The PELE-Seq method is ideal for investigating the dynamics of rare alleles in a broad range of reduced-representation sequencing methods, including targeted amplicon sequencing, RAD-Seq, ddRAD, and GBS. PELE-Seq is also well-suited for whole genome sequencing of mitochondria and viruses, and for high-throughput rare mutation screens.

**Electronic supplementary material:**

The online version of this article (doi:10.1186/s12864-016-2669-3) contains supplementary material, which is available to authorized users.

## Background

Populations with high levels of genetic heterogeneity are able to evolve rapidly through natural selection, for example providing the basis for drug resistance in populations of microbes, viruses, and tumor cells [1–3]. In order to understand how these heterogeneous populations evolve in response to selection, it is important to be able to characterize the full catalog of genetic variation present in the population, including *de novo* mutations and minor alleles.

The reduced cost of DNA sequencing has powered the wide-scale discovery of functional and disease-causing single nucleotide polymorphisms (SNPs) and genomic regions under selection [4, 5]. However, the current high error rate (~1 %) leads to the generation of millions of sequencing errors in a single experiment. Thus, when attempting to sequence *de novo* mutations or genetically heterogeneous populations, it is challenging to distinguish between errors and true rare genetic variants [6–9]. Errors are also introduced into NGS data during the PCR amplification step of library generation and during library preparation when acoustic shearing is used to fragment the DNA molecules [10–12].

Here we present a new method of rare allele detection that removes sequencing and PCR errors from deep-sequencing NGS data without a loss of sensitivity, called Paired-End Low-Error Sequencing (PELE-Seq). The PELE-Seq method is based on two principles. First, each DNA molecule is prepared with a short insert size and then sequenced with overlapping paired-end (PE) reads. The reads are then merged into a single, high-quality consensus sequence that is free of sequencing errors. Second, during library generation each sample is PCR amplified with a mixture of two uniquely barcoded adapters that attach to the same end of the DNA molecules. The PELE-Seq analysis pipeline incorporates the dual-barcoding information to increase the sensitivity of the method by reducing the incidence of false-positive SNPs in the sequencing data.

Sequencing error reduction through the use of overlapping read pairs (ORPs) has been described previously by Chen-Harris et al., who showed that the use of overlapping paired-end reads dramatically reduces the occurrence of sequencing errors in NGS data [11]. Their group reported that when overlapping paired-end reads are merged to remove sequencing errors, a low level of background error remains in the data that is presumably due to PCR error. This background error rate can be empirically calculated using the ORP method by sequencing a pure sample and counting the errors that remain after mismatched nucleotides are removed from the overlapped reads. PELE-Seq improves on the ORP method by incorporating a dual-barcoding system that reduces background errors in the data, allowing for a more sensitive detection of rare polymorphisms.

To test the performance of the PELE-Seq method, we generated a series of control *E. coli* “spike-in” DNA libraries containing known rare SNPs at various allele frequencies. The libraries were created through the serial dilution of DNA from the E. coli K12 substrain W3110 into DNA from the *E. coli* B substrain Rel606. The K12 W3110 substrain of *E. coli* contains a SNP every ~117 bp compared to *E. coli* B substrain Rel606 [13, 14]. The spike-in DNA mixtures contained rare SNPs at average allele frequencies ranging from 0.22–0.42 % of total nucleotides at a position. We tested the effectiveness of the PELE-Seq, ORP, and standard DNA-Seq methods at identifying the expected rare SNPs using ultra-deep sequencing at various read depths ranging from 43,000–60,000× coverage of raw reads. We show that PELE-Seq can detect rare alleles with 100 % specificity and without a loss of sensitivity compared to standard methods.

We applied the PELE-Seq method to sequence rare alleles in a wild population of *Caenorhabditis remanei* nematode worms. *C. remanei* are highly heterogeneous, non-hermaphroditic nematode worms that are amenable to studies investigating the genetic basis of the response to natural selection [15]. In this study, we sampled the genome of an ancestral (wild) population originating from 26 wild mating pairs from Toronto, Ontario that were lab-propagated for a total of 23 generations. We show that PELE-Seq can detect changes in the rare allele frequencies between the genomes of the wild and lab-adapted populations, including SNPs that appear in one population but are completely absent in the other, using an overlapped paired-end (OPE) read depth of 900× per population.

## Results

### PELE-Seq library preparation and data analysis

**Fig. 1:**
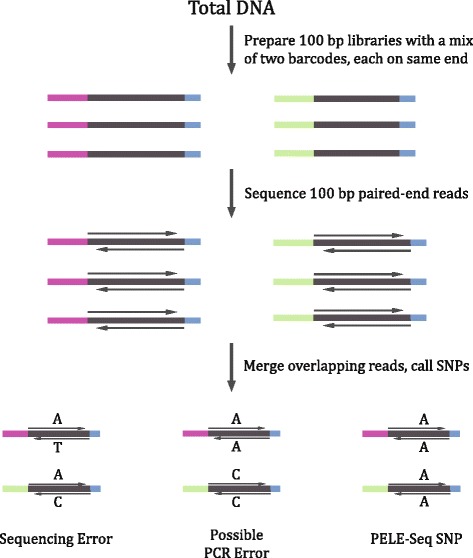
The PELE-Seq method of rare variant calling. DNA libraries with a 100 bp insert size are paired-end sequenced using 100 bp reads, generating an overlap region of approximately 100 bp. The overlapping reads are merged into a consensus sequence and mismatching bases are discarded. A mixture of two separately-barcoded P1 adapters (*green and purple*) is ligated to each sample. The P2 adapter that is common to all DNA molecules is shown in *blue*. In order to pass PELE-Seq quality filtering, SNPs must be present in both paired-end reads and with both barcodes

PELE-Seq improves the specificity of standard SNP-calling methods by reducing the occurrence of false-positive SNPs in NGS data. An overview of the PELE-Seq method is illustrated in Fig. 1. PELE-Seq library preparation and analysis involves two separate error-filtering strategies which are combined during analysis:

1. Overlapping Read Pairs (ORPs)

Illumina 100 bp paired-end sequencing of short 100 bp DNA inserts is used to generate two completely overlapping paired-end reads from each DNA molecule. The overlapping paired-end reads are then merged into one high-quality consensus sequence. After trimming off the overhanging bases and filtering for high quality scores (Q ≥ 60, as calculated by the program SeqPrep), the resulting consensus sequence has a much lower incidence of false positive SNPs compared to the non-overlapped reads.

2. “Dual-barcoding” System

A “dual-barcoding” system is used to increase the sensitivity of rare variant detection by removing PCR errors from the data. The barcoding system employed by PELE-Seq works by attaching two independently-barcoded adapters to each sample, with the barcodes on the same end of each DNA insert. The barcode information is used to filter out SNPs that are called with only a single barcode, which are putative PCR errors.

PELE-Seq data analysis incorporates information from both the barcoding and the overlapping steps, to produce a list of very high quality SNPs that have passed numerous quality control filters without a loss in sensitivity. Rare alleles are called using the program LoFreq, which calls rare variants using a Bonferroni-corrected *P*-value threshold of 0.05 [16]. We’ve empirically found that altering the variant-calling parameters used to call SNPs, such as allele frequency cutoffs and Q scores, can lead to very different SNP calls for a given sequencing library. The PELE-Seq dual barcoding system allows for an additional round of SNP-calling on the separately barcoded files, using less-stringent parameters than those that are required for calling SNPs from reads with both barcodes combined. Counting the SNPs that appear in both separately-barcoded libraries leads to an increase in the sensitivity of SNP-calling compared to the ORP method alone.

The PELE-Seq analysis pipeline works by creating two lists of SNP calls for each library: List A contains SNPs called from the merged overlapping reads (ORP data) and List B contains the SNPs that appear in both individually-barcoded libraries, using less stringent parameters to call variants from the overlapping reads. The final list of PELE-Seq SNP calls is created by adding Lists A and B.

### PELE-Seq specificity and sensitivity

We first sought to empirically determine the specificity and sensitivity of the PELE-Seq variant calling method. We PELE-sequenced four control *E. coli* DNA “spike-in” mixtures containing SNPs present at average frequencies ranging from 0.22–0.42 % (Table 1). We identified 64 expected “true-positive” control SNPs by sequencing the pure *E. coli* K12 substrain W3110 at 2000× raw read depth and aligning it to the genome of the pure *E. coli* B substrain Rel606 (Additional file 1). The purity of the original DNA samples was verified through sequencing by aligning the sequencing reads from both strains to the Rel606 genome. The “true positive” SNPs were found to be present at 100 % frequency in the W3110 DNA, and all other positions contained reference bases at 100 % frequency. Similarly, the Rel606 DNA contained a single nucleotide at each position in the genome (Additional file 2). The genome space sequenced was reduced to 14 Kb by using Restriction-site Associated DNA Sequencing (RAD-Seq) to sequence only the 200 nucleotides adjacent to an SbfI restriction enzyme cut site [17]. SbfI cuts the sequence CCTGCAGG, which occurs ~70 times in the *E. coli* genome.

**Table 1:** Allele frequencies of rare SNPs in control *E. coli* “spike-in” DNA mixtures

Library	Total read depth	Allele frequency
1	38,000	0.0042
2	38,000	0.0025
3	71,000	0.0022
4	66,000	0.0020

Four control “spike-in” DNA mixtures were created by serial dilution of one *E. coli* substrain (W3110) into another (Rel606). The libraries were PELE-sequenced to an average total read depth of 53,000× OPE. The rare alleles detected in the control libraries had average allele frequencies ranging from 0.20–0.42 % or 1/238-1/500 of total reads

**Table 2:** Rare SNPs identified using the PELE-Seq, ORP, and standard DNA-Seq methods, at various read depths

Average read depth per barcode	PELE positives	PELE false positives	ORP positives	ORP false positives	Standard positives	Standard false positives
1000	13	0	6	0	6	2
5000	19	0	18	0	24	7
10000	42	0	37	0	36	12
15000	36	0	32	0	35	13
18000	40	0	35	0	41	42

A control spike-in library containing 64 expected rare alleles present at 0.42 % frequency was sequenced with the PELE-Seq, ORP, and Standard DNA-Seq methods at various read depths. The read depths listed are for the overlapping paired-end (OPE) reads per barcode of the PELE-Seq libraries. The methods are compared using the same number of raw reads, such that the standard DNA-Seq bam files have a read depth that is 2.4× that of the PELE-Seq bam files (2400–43,000× per barcode), to account for the loss associated with merging overlapped reads to create ORPs

**Fig. 2:**
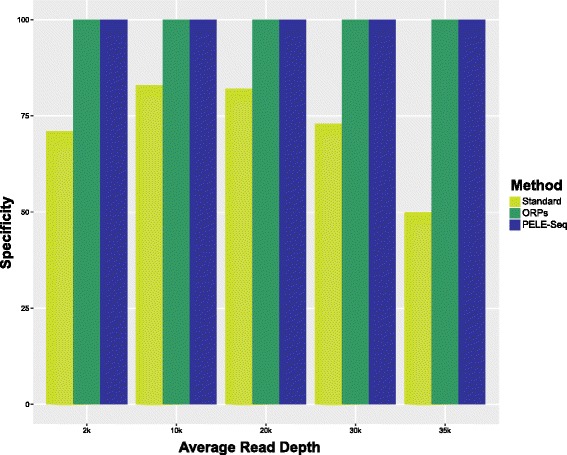
The PELE-Seq and ORP methods detect rare alleles with 100 % specificity. Sequencing a control *E. coli* DNA library containing 64 expected rare SNPs present at 0.42 % average allele frequency, with read depths ranging from 2000–35,000× OPE (4800–88,000× non-overlapped read depth), produces 100 % specific data with PELE-Seq and ORP methods, compared to the 50–77 % specificity achieved with standard (non-overlapped) sequencing methods. Standard DNA-Seq of the control libraries resulted in 12 false positive mutations, compared to zero for the PELE-Seq and ORP methods. The methods were compared using the same number of raw reads

**Fig. 3:**
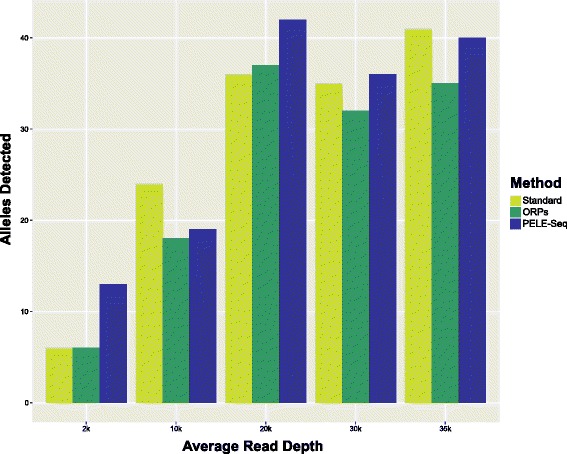
PELE-Seq is more sensitive than the ORP and standard DNA-Seq methods at detecting rare SNPs. The PELE-Seq method detects a similar number of rare alleles present at 0.42 % average allele frequency as the standard DNA-Seq method, and is more sensitive than the ORP method. A control *E. coli* DNA library containing 64 expected rare SNPs present at 0.42 % average allele frequency was sequenced with read depths ranging from 2000–35,000× OPE (4800–88,000× non-overlapped read depth). The methods were compared using the same number of raw reads

The control spike-in libraries were sequenced to a total read depth of ~18,000–30,000× overlapping paired-end (OPE) reads per barcode. To test the effectiveness of the method at various depths of coverage, unsorted sam files were truncated to depths of 1000 × -20,000× OPE per barcode. In addition, various allele frequency and quality score cutoffs were tested to optimize rare variant identification with the method (Additional file 3). The PELE-Seq and standard DNA-Seq libraries were compared using the same number of raw reads, such that the standard DNA-Seq bam files used to call SNPs have a read depth that is 2.4× that of the PELE-Seq bam file, to account for the loss associated with merging overlapped reads to create ORPs. We found that PELE-Seq and ORP data had no false positive SNP calls, compared to 50–80 % specificity achieved by sequencing the same raw reads using standard DNA-Seq methods (Fig. 2 and Table 2). The sensitivity of the PELE-Seq method was not significantly different from standard DNA-Seq data when using the same raw sequencing reads, but was consistently more sensitive than data generated with the ORP method alone. For our sequencing libraries, the optimal read depth tested was 10,000× OPE per barcode (Fig. 3).

**Fig. 4:**
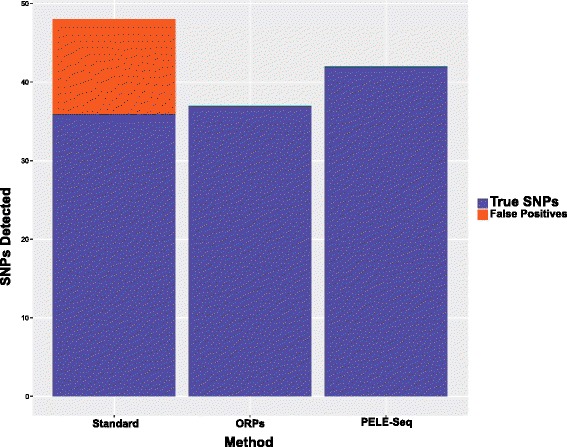
PELE-Seq data has zero false positive SNPs and high sensitivity. Sequencing a control *E. coli* DNA library containing 64 expected rare SNPs present at 0.42 % average allele frequency with PELE-Seq at 20,000× OPE read depth (48,000× raw read depth) produces 100 % specific data, compared to 75 % specificity achieved with standard sequencing methods. Standard DNA-Seq of the control libraries resulted in 12 false positive mutations, compared to zero with the PELE-Seq method

**Fig. 5:**
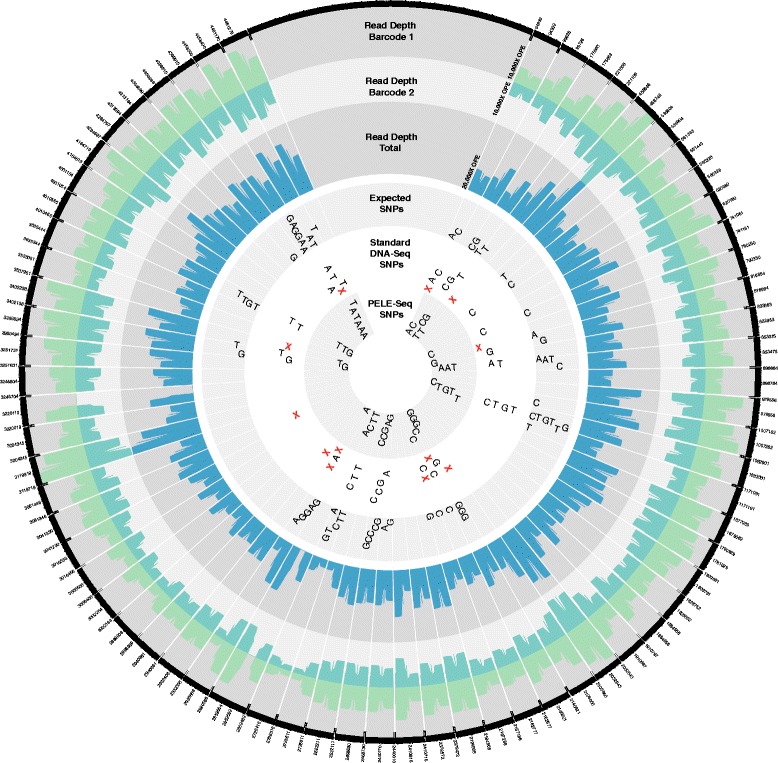
Rare SNPs present at 0.42 % frequency, detected with PELE-Seq and standard DNA-Seq methods. A control *E. coli* library containing rare alleles present at 0.42 % frequency were sequenced with PELE-Seq and standard DNA-Seq with 20,000× OPE depth (48,000 non-overlapped read depth). The read depths of the individual barcode files are plotted in *light green*, and the total read depth is plotted in *blue*. The SNPs detected with PELE-Seq are plotted in the inner circle, and the standard DNA-Seq SNPs are plotted in the next outer circle. The 12 false positive SNP calls present in the standard DNA-Seq data are designated with a red “X”. Of the 64 expected rare SNPs, PELE-Seq detected 42 SNPs with 100 % specificity, compared to 36 SNPs with 75 % specificity achieved with standard DNA-Seq methods

When detecting rare SNPs present at 0.4 % average allele frequency, with 10,000× OPE read depth per barcode, PELE-Seq can identify 42 of the expected 64 SNPs with 100 % specificity, compared to 36 SNPs with 75 % specificity that is achieved with standard DNA-Seq (Figs. 4, 5 and Additional file 4). The remaining 22 SNPs were undetectable without compromising the specificity of the method. By setting a very low allele frequency cutoff (≤0.001) to call SNPs, 53 true positive SNPs were identified, but 108 false positive SNP calls were also made using those parameters (Additional file 3). Upon further investigation, we found that the remaining uncalled SNPs were present at far below the expected frequency of 0.4 % in the libraries, rendering them indistinguishable from background PCR errors. The reason for the low frequency of these 11 alleles in the original spike-in libraries is unclear, but may be due to stochastic bias occurring during PCR amplification or serial dilution, or perhaps the GC-bias of NGS data. Regardless, this lack of detection is not an issue with the sensitivity of the PELE-Seq variant calling method, and future improvements in amplification-free and unbiased sequencing methods should improve the detection of all rare alleles. For PELE-Seq studies that seek to identify rare alleles with 100 % sensitivity, we recommend sequencing multiple replicates of each sample, each with two barcodes and with 10,000× OPE read depth per barcode.

At read depths below 5,000× OPE per barcode, the PELE-Seq and Standard DNA-Seq methods were only able to identify 13/64 and 6/64 of the expected SNPs in the 0.4 % AF libraries, respectively. In addition, SNPs with very low allele frequencies (≤0.25 %) were extremely challenging to distinguish from the background PCR errors in the libraries. Only 14/64 (PELE-Seq) and 13/64 (Standard DNA-Seq) of the expected SNPs at 0.25 % were detected with a read depth of 35,000× OPE per barcode. These SNPs were detected with 100 % specificity with PELE-Seq (Additional file 3).

**Fig. 6:**
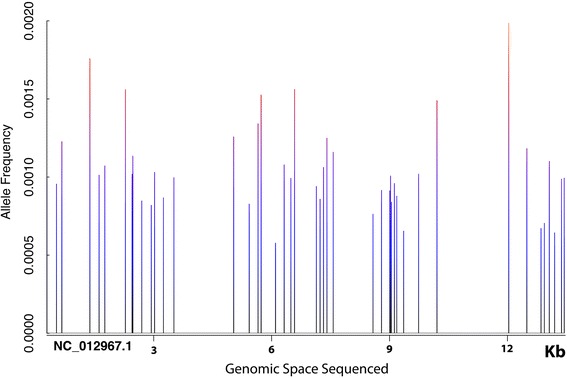
Background PCR errors are found in distinct clusters throughout the sequenced RAD tags. ORP libraries sequenced to 10,000× OPE depth contained 109 false positive mutations when SNPs were called with Lofreq using default parameters without a minimum allele frequency cutoff above the level of background error. These mutations appeared in distinct clusters throughout the sequenced RAD tags. The SNPs are plotted across the 14 Kb of sequenced RAD tags. Each blue bar represents a cluster of 2–3 errors. Of the 140 RAD tags sequenced, only 45 contained PCR errors, and each of those contained an average of 2.6 PCR errors. The maximum allele frequency of the sequencing errors was 0.002 at this read depth

**Fig. 7:**
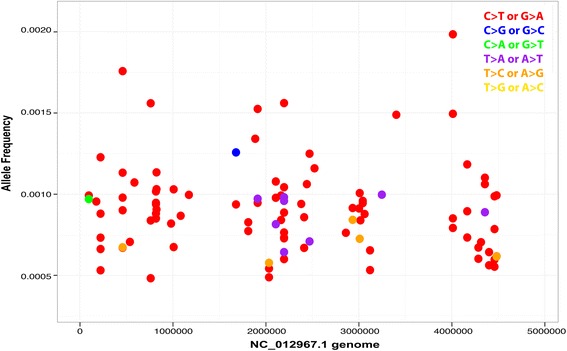
Background PCR errors are predominately C > T transitions. ORP libraries sequenced to 10,000× OPE read depth contained 109 false positive mutations when SNPs were called with Lofreq using default parameters without a minimum allele frequency cutoff above the level of background error. These mutations were found to be overwhelmingly C > T transitions, when classified based on the mutated pyrimidine of each base pair

False-positive SNP calls were generated when overlapping paired-end data was not filtered with a minimum allele frequency threshold that was above the level of the background error rate. Overlapping paired-end read libraries sequenced to 10,000× OPE depth contained 109 false positive SNPs when rare variants were called with Lofreq using default parameters with no minimum allele frequency cutoff (Additional file 5). These errors appeared in distinct clusters throughout the genome and were found to be overwhelmingly C > T transitions, when classified based on the mutated pyrimidine of each base pair (Figs. 6 and 7). C > T transitions are a relatively common mutational event caused by spontaneous deamination of 5-methly-cytosine [18, 19]. C > T transitions have previously been reported to comprise the majority of PCR errors in NGS data [20].

We’ve found that in order to remove false-positive SNP calls from overlapping paired-end read data without a drop in sensitivity, it is critical to set a minimum allele frequency cutoff that is above the maximum per-sequenced-base error rate of a library. The background PCR error rate of PELE-Seq data can be calculated by sequencing a pure DNA control sample with overlapping read pairs, and then counting the unaligned nucleotides that remain in the data after the sequencing errors are removed through merging the overlapping reads. In practice, the most important metric of background error for calling SNPs is not the overall error rate in the data, but rather the maximum error rate per-sequenced-base according to genome position, as this correlates to the allele frequency of the errors. In other words, sequencing data may have a very low overall incidence of PCR errors, but certain positions may can have an unusually high rate of error, which is difficult to distinguish from true SNPs. Because this error rate is impossible to calculate *a priori*, we recommend that PELE-Seq projects include a control amplicon containing a known rare allele, that is run alongside the experimental samples in order to empirically determine the optimal SNP calling parameters for each library. By sequencing a control amplicon, the SNP results can be optimized for each unique library and sequencing depth to ensure high-quality SNP calls with 100 % specificity and high sensitivity.

We’ve determined the optimal parameters for calling SNPs from our spike-in libraries at various read depths using the PELE-Seq, ORP, and standard DNA-Seq methods, which are reported in Additional file 6. For our libraries, the minimum SNP allele frequency cutoff of ≥0.002 was found to eliminate all false positive mutations in the overlapping read data when read depths above 1,000× OPE per barcode are used (Additional file 3). We’ve empirically found that libraries sequenced with lower read depths have a higher effective per-sequenced-base error rate, as they require more stringent allele frequency filtering to achieve 100 % error-free data. This implies that the effective background error rate for a library is dependent on depth of coverage.

### Detection of rare and *de novo* mutations in wild and lab-adapted *C. remanei*

We applied PELE-Seq to track changes in the rare allele frequencies of a wild population of *C. remanei* nematode worms that was subjected to laboratory-adaptation. The ancestral (wild) *C. remanei* population originated from 26 mating pairs of nematodes that were expanded to a population of 1000+ individuals and then frozen within three generations. A branch of this ancestral population was grown in the lab for 23 generations, during which time it was culled randomly to a population of 1000 individuals for each generation. The lab-adapted population was also subjected to 2 freezes and 9 bleach treatments (hatchoffs) during this time. The numerous selection events endured by the lab-reared nematodes were expected to lower the genetic diversity of the population via drift and bottlenecking. Rare advantageous SNPs could also be selected for during the process of lab-adaptation.

To assess the changes in genetic diversity of the nematode population before and after lab-adaptation, DNA from the wild and laboratory-adapted populations of *C. remanei* worms was PELE-sequenced using PacI RAD-Seq. The PacI restriction enzyme cuts the sequence AATTAATT, which occurs 2044 times in the *C. remanei* caeRem3 genome. In order to further decrease the complexity of the genome, we performed an additional restriction enzyme digestion with NlaIII to destroy a portion of the RAD tags in the library. NlaIII cuts the sequence CATG, which is within the sequence of approximately 30 % of the PacI RAD tags. The resulting genome space covered was approximately 300 Kb, which was sequenced to an average of 2000× OPE read depth.

**Fig. 8:**
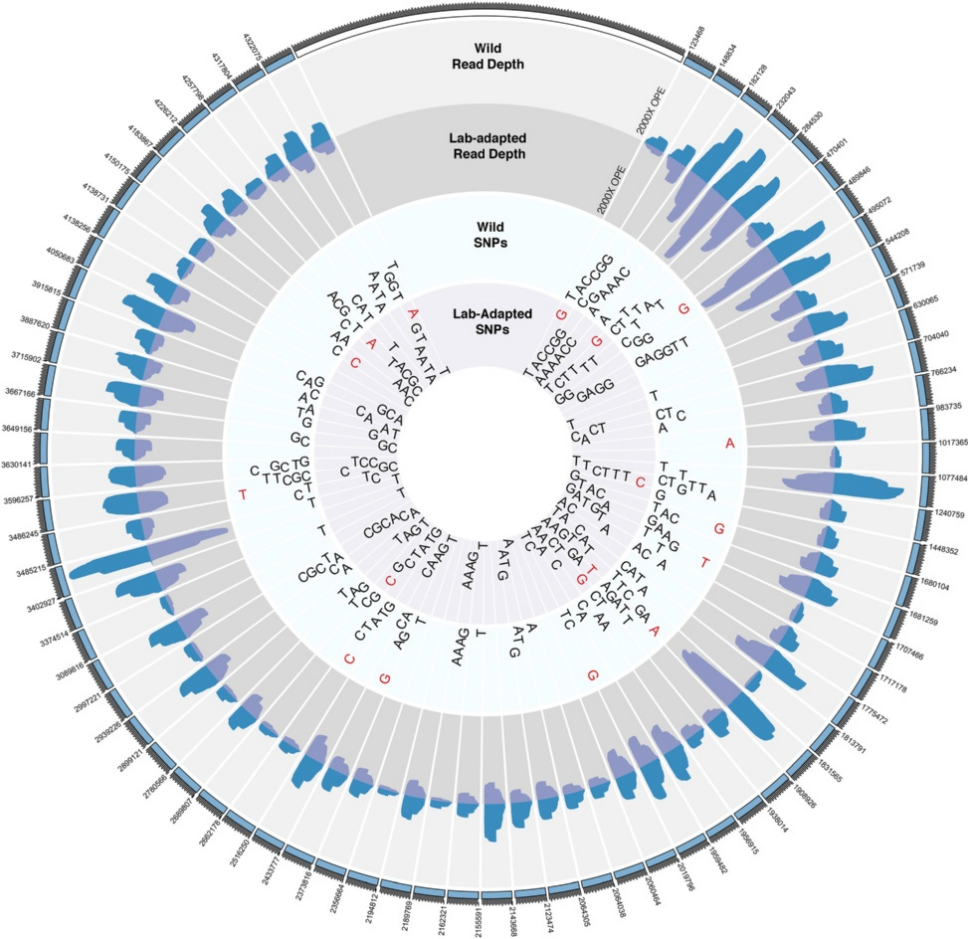
Wild and lab-adapted *C. remanei* populations have distinct SNP profiles. SNPs detected in the *C. remanei* population before and lab-adaptation are plotted for a subset (0.006 %) of the caeRem3 genome, sequenced at 2000× OPE depth. SNPs detected with PELE-Seq in the wild population are plotted in the *light blue circle*; SNPs detected in the lab-adapted population are plotted in the inner *light purple circle*. SNPs present in both the wild and lab-adapted populations are shown with *black letters*. SNPs appearing in only the wild or lab-adapted populations are shown with *red letters*

**Fig. 9:**
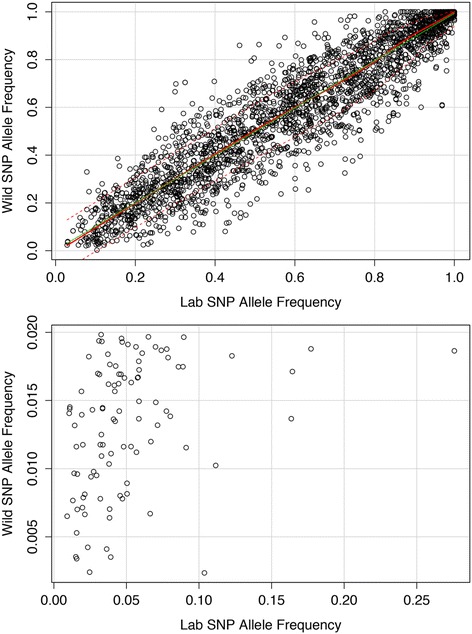
SNP allele frequencies in *C. remanei* before and after lab-adaptation. The allele frequencies of 13,000 SNPs that are present in both populations are plotted, with each point representing a SNP in the genome. *Top* Allele frequencies before and after lab-adaptation for all SNPs that are present in both populations, detected with PELE-Seq. SNPs in the top left corner are less frequent in the lab-adapted worms; SNPs in the bottom right corner are more frequent in the lab-adapted worms. The estimated 0.25 and 0.75 quantiles of the square root of variance are shown with the dashed red lines. *Bottom* A zoom-in of allele frequencies before and after lab-adaptation, for SNPs present below 2 % in the wild *C. remanei* population. Seven rare SNPs in the wild population increased in frequency at least fivefold after lab adaptation

With PELE-Seq we found that the wild and lab-adapted *C. remanei* populations had a distinct profile of SNPs before and after laboratory-adaptation (Fig. 8). By plotting the allele frequencies of SNPs present in both populations before and after lab adaptation, it is possible to visualize the changes in the allele frequencies of minor alleles in a population undergoing a response to selection (Fig. 9). We identified rare SNPs in the wild *C. remanei* populations whose allele frequencies increased dramatically during lab-adaptation (Additional file 7). Table 3 lists 7 rare SNPs found in the wild population that increase in frequency at least five-fold in the lab-adapted population.

**Table 3:** Rare SNPs in the wild *C. remanei* population that significantly increase in frequency after lab-adaptation

Position	Ref	Alt	Wild allele frequency	Wild allele depth	Lab allele frequency	Lab allele depth
2933656	G	A	0.010233	11	0.111517	61
84255709	C	A	0.018789	27	0.17717	149
89350272	A	G	0.018276	60	0.122693	379
114867644	G	A	0.017125	43	0.164325	386
127723967	C	G	0.00235	31	0.103739	738
138506868	A	C	0.013662	31	0.163548	236
141293514	T	C	0.018643	25	0.275908	205

Seven SNPs found below 2 % frequency in the wild *C. remanei* population increased in frequency at least five-fold in the lab-adapted population*.* The read depths listed are those of the detected rare allele, not the total read depth at that position. Read depths listed are for overlapping paired-end (OPE) reads

**Fig. 10:**
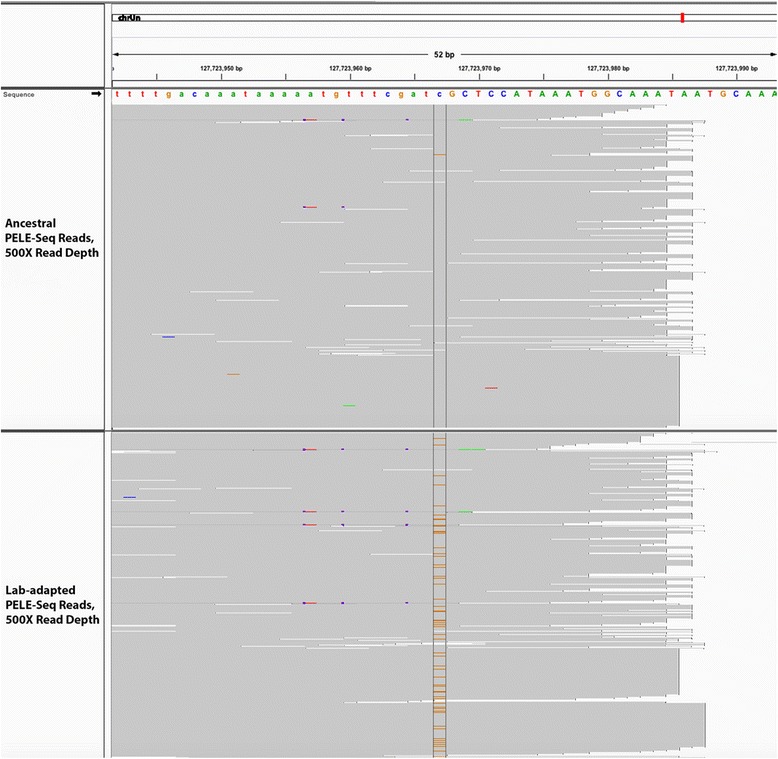
A SNP near the promoter region of *ugt-5* increases in frequency 44× after lab adaptation. A G > C transversion found at below 1 % frequency in the ancestral *C. remanei* population has a 44× increase in frequency after 23 generations of laboratory adaptation. This SNP maps to the promoter region of a gene predicted to be homologous to the *C. elegans* gene *ugt-5*, which codes for an enzyme responsible for the removal of drugs, toxins, and foreign substances. The *top panel* shows 500 sequencing reads from the ancestral (wild) population; the *bottom panel* shows 500 sequencing reads from the lab-adapted population. The non-reference SNP at position 127,723,967 of the caeRem3 genome is visible in *orange*

We detected a SNP at position 127,723,967 of the caeRem3 (WUSTL) genome that had increased in frequency by 44× in the lab-adapted population. The number of reads containing this G > C transversion jumped from 31/13000 (0.24 %) in the wild population to 738/7100 (10.4 %) in the lab-adapted population. This SNP is located upstream of the promoter region of a gene predicted by the UCSC Genome Browser to be homologous to the *C. elegans* gene *ugt-5*, which codes for a UDP-Glucuronosyltransferase. The read pileups mapping to this SNP are shown in Fig. 10.

We then sought to determine if PELE-Seq could detect SNPs present in one population that were completely absent in the other. The high level of sequencing error in standard NGS libraries typically prevents any investigation into the presence of “*de novo*” alleles, as ultra-deep NGS data contains sequencing errors at every position which are impossible to distinguish from true SNPs. In order to call SNPs that are present in one population but completely absent in another, we used a variant calling program that is specifically designed to detect rare somatic mutations present in one sample and completely absent from another, which is Seurat Somatic [21]. Seurat Somatic was designed to take two separate bam files as input and compare them to each other when searching for rare SNPs. The program outputs SNP calls that are present in one sample but completely absent in the other. We refer to these SNPs as “putative *de novo* SNPs” since they are undetectable in the original population when sequenced with high read depth. It is important to ensure that the SNPs called using this method are completely absent from the wild population, as a false-negative SNP call in the wild population would appear as a false positive *de novo* mutation in the lab-adapted population.

We’ve identified 91 rare SNPs that are present in the lab-adapted population but are undetectable in the wild population, using a minimum read depth of 900× OPE (Additional file 7). Many of these putative *de novo* SNPs were present at significant frequencies (5–15 %) in the lab-adapted population, despite being absent in the wild population. Table 4 contains a list of 9 putative *de novo* SNPs found to be present above 6 % in the lab-adapted population. The read pileups at these positions provide strong supporting evidence that the SNPs are completely absent in the wild DNA reads and are therefore present below 0.11 % in the libraries (Additional file 7).

**Table 4:** Putative *de novo* SNPs present in the lab-adapted *C. remanei* population above 6 %

Position	Ref	Alt	Wild allele frequency	Lab allele frequency	Wild depth	Lab depth
8678151	T	C	0	0.07	998	993
22410779	C	T	0	0.16	992	997
23864162	T	A	0	0.06	991	895
27788600	A	G	0	0.069	995	956
67266085	C	T	0	0.066	998	846
67492174	A	G	0	0.07	940	961
96566683	T	C	0	0.071	988	971
127028996	C	T	0	0.065	982	965
143968069	T	G	0	0.121	996	988

Many putative *de novo* SNPs were present at significant frequencies in the lab-adapted population, despite being absent in the wild population. Using a minimum read depth of 900× overlapping paired-end (OPE) reads, PELE-Seq detected 9 putative de novo SNPs found above 6 % frequency in the lab-adapted population

**Fig. 11:**
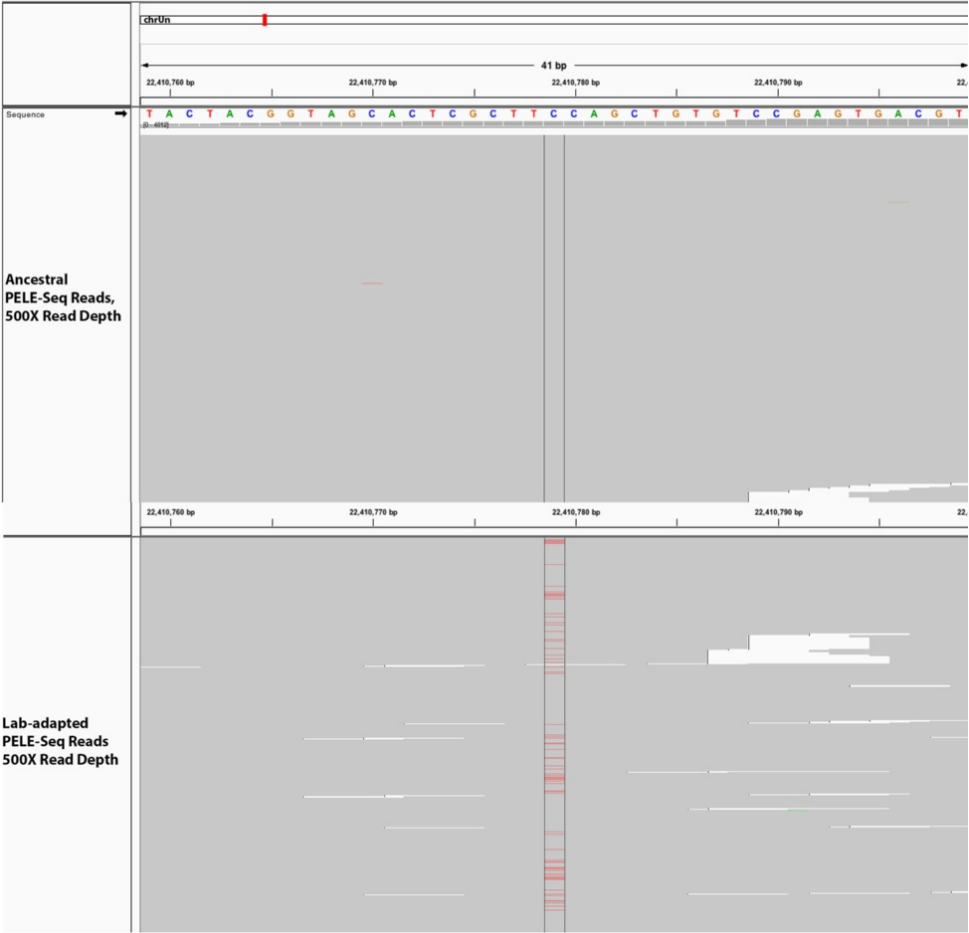
An *ilrd-14* SNP at 16 % frequency in the lab-adapted and 0 % in the wild *C. remanei*. A C > T transition that is completely undetectable in the wild population (0/992 reads) is present at 16 % frequency in the lab-adapted population (159/997 reads). This SNP is located within an intron of a gene predicted to be homologous to the *C. elegans* gene *ilrd-14*, which codes for an insulin/EGF receptor L-domain protein. The *top panel* shows 500 sequencing reads from the ancestral (wild) population; the *bottom panel* shows 500 sequencing reads from the lab-adapted population. The non-reference SNP at position 22,410,779 of the caeRem3 genome is visible in *red*

**Fig. 12:**
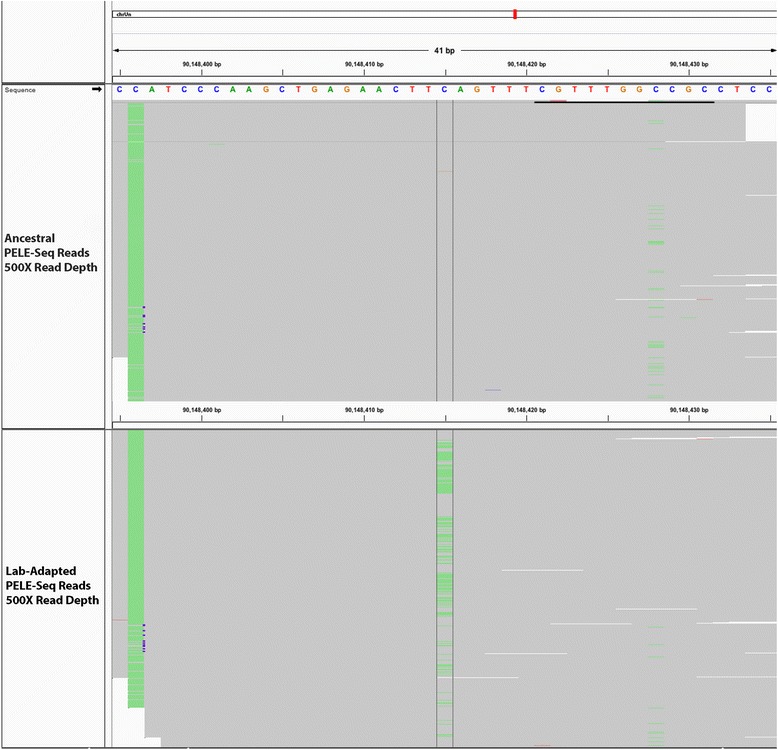
A SNP near *srh-265* at 21.5 % in the lab-adapted and 0 % in the wild *C. remanei*. When a minimum read depth of 800× OPE was used to detect putative *de novo* alleles, a C > A transversion was found that increased from 0/862 reads in the wild population to 153/811 reads (21.5 %) in the lab-adapted population. This SNP is upstream of a gene predicted by UCSC to be homologous to the *C. elegans* gene *srh-265*, which codes for a serpentine receptor, of class H. The *top panel* shows 500 sequencing reads from the ancestral (wild) population; the *bottom panel* shows 500 sequencing reads from the lab-adapted population. The non-reference SNP at position 90,148,415 of the caeRem3 genome is visible in *red*

We identified a SNP at position 22,410,779 of the caeRem3 genome that is completely absent in the wild population (0/992 reads) and is present at 16 % frequency in the lab-adapted population (159/997 reads) (Fig. 11). This SNP is located within an intron of a gene predicted by UCSC to be homologous to the *C. elegans* gene *ilrd-14*, which codes for an insulin/EGF receptor L-domain protein. In addition, when a minimum read depth of 800× OPE was used to detect putative *de novo* alleles, a SNP at 90,148,415 was found to increase from 0/862 reads in the wild population to 153/811 reads (21.5 %) in the lab-adapted population (Fig. 12). This SNP is upstream of a gene predicted by UCSC to be homologous to the *C. elegans* gene *srh-265*, which codes for a serpentine receptor, of class H.

**Fig. 13:**
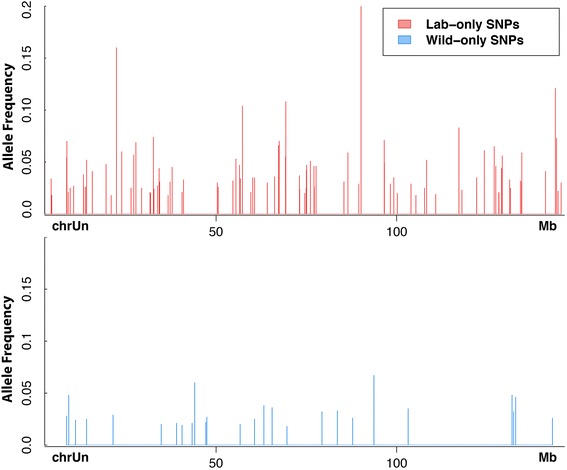
Putative *de novo* SNPs are more numerous than SNPs lost during lab-adaptation of *C. remanei*. With PELE-Seq we identified 91 putative *de novo* mutations that are found only in the lab-adapted *C. remanei* population (*red*), and 19 SNPs found only in the wild population (*blue*), using a minimum read depth of 900× OPE. Each vertical line represents a single SNP and the height of the line is proportional to the allele frequency. The SNPs present only in the lab-adapted population occur more frequently (91 vs. 19 SNPs) and with higher allele frequencies (max 16 % vs 6 %) than the SNPs present only in the wild population

We then used Seurat Somatic to identify 19 SNPs that were present in the wild population but were undetectable in the lab-adapted population, using a minimum read depth of 900× OPE (Additional file 7). These SNPs were all present at frequencies below 6 % in the wild population and were presumably lost due to bottlenecking and genetic drift. These SNPs appeared at a lower rate and with lower allele frequencies than the putative *de novo* SNPs appear in the lab-adapted population (Fig. 13).

## Discussion

Current genomic studies of genetically heterogeneous samples, such as *de novo* mutations in growing tumors or natural populations that are difficult to sequence as individuals, are hampered by the difficulty in distinguishing alleles at low frequency from the background of sequencing and PCR errors. We have developed a method of rare allele detection that mitigates both sequence and PCR errors, called PELE-Seq. PELE-Seq was evaluated using synthetic *E. coli* populations and used to compare a wild *C. remanei* population to a lab-adapted population. Our results demonstrate the utility of the method and provide guidelines for optimal specificity and sensitivity when using PELE-Seq.

The PELE-Seq method was developed in order to investigate the behavior of rare alleles within dynamic, heterogeneous populations. Examples of research applications that the PELE-Seq method is well-suited include: RAD and double-digest RAD ddRAD [22] sequencing of pooled populations, whole genome sequencing of populations with small genomes such as viruses and mitochondria, targeted-amplicon DNA capture of tumors and populations, and high-throughput rare mutation screens such as the TILLING by sequencing method [23]. The PELE-Seq method could also be useful for reduced-representation sequencing studies using barcoded individual organisms if the goal is to uncover rare genetic variants within an individual, such as somatic and mitochondrial mutations. In this instance, each individual would need to have two separate barcodes and would need to be sequenced to a high read depth in order to detect rare alleles.

Many phenotypic traits are believed to arise from combinations of large numbers of rare genetic variants. For example, modern human Genome-Wide Association Studies (GWAS) are currently unable to identify the expected number of disease-associated alleles in humans based on heritability studies. This may be due to the fact that the disease-causing rare alleles are present at frequencies below the limit of detection with standard sequencing methods, leading researchers to propose that many inherited diseases could result from unique combinations of rare susceptibility genes [24, 25]. PELE-Seq would be a very useful tool for studies seeking to understand the roles of rare mutations within a population.

By using PELE-Seq, we increased the number of independent validations of a rare SNP by sequencing each molecule twice with overlapping paired-end reads and by calling each SNP twice through the use of multiple barcodes. The multiple PELE-Seq quality control steps result in genotype calls of low-frequency alleles with a false positive rate of zero, allowing for the specific detection of rare alleles in genetically heterogeneous populations. For our libraries, we found that the optimal level of read depth was 10,000× of overlapping paired-end (OPE) reads per barcode. Sequencing below this level reduced the sensitivity of the method, while sequencing above this level, up to the maximum tested depth of 18,000× OPE per barcode, did not significantly improve the results.

We found that calling rare variants from overlapped reads without filtering with a minimum allele frequency cutoff led to the introduction of false-positive genotype calls in the sequencing data. These errors are believed to be caused during PCR amplification, and are predominately C > T transversions, when classified based on the pyrimidine of the base pair. C > T transversions that are present in NGS data due to PCR error have been previously reported [20]. We have demonstrated that these erroneous base calls can be removed by carefully selecting optimized parameters during variant calling, so that the minimum allele frequency cutoff used to call SNPs is above the maximum per-sequenced-base error rate of a library.

Because the background error rate for a library is impossible to calculate *a priori*, we recommend that PELE-Seq projects include a control amplicon containing a known rare allele be run alongside the experimental samples, in order to empirically determine the optimal SNP-calling parameters for each library. Running a small amplicon as a control would be relatively inexpensive and the extra sequencing cost would be well-worth the improved specificity and sensitivity of the sequencing data. If it unfeasible to sequence a control amplicon for a PELE-Seq project, the optimized SNP-calling parameters that we’ve identified for calling rare alleles from our libraries at various read depths could theoretically be generalized to other libraries that are prepared in the same way.

Sequencing error reduction through the use of overlapping read pairs (ORPs) has been described previously by Chen-Harris et al., who show that the use of overlapping paired-end data dramatically reduces the occurrence of sequencing errors in NGS data [11]. Their group concluded that PCR error is the dominant source of error for Illumina sequencing data with a quality score above Q30, which they estimate to be on the order of 0.05 %. PELE-Seq improves on the ORP method by incorporating a dual-barcoding system that allows for the removal of PCR errors from the data. We have shown that the PELE-Seq method is more sensitive than the ORP method for detecting rare alleles.

The main disadvantage of the PELE-Seq and ORP methods compared to standard, non-overlapped NGS data is that the sequencing cost is ~2.4 times more for a given amount of genomic space, due to the use of overlapping paired-end reads. Also, due to the high depth of read coverage (~20,000× OPE) that is required to detect most alleles present below 0.5 %, using the PELE-Seq method can lead to high sequencing costs for some projects. The number of genetic markers or amplicons to sequence should be carefully chosen, depending on the specific goals of each research project. The exact cost of sequencing PELE-Seq libraries depends on the accuracy of the size-selection process during library preparation, as any overhanging bases are trimmed off of the read pairs during analysis. Sequencing methods such as ddRAD and Genotyping By Sequencing (GBS) that generate a uniformly-sized library should experience negligible losses of sequencing data during the overlapping step, and in that case the cost of PELE-Seq would be exactly twice that of standard sequencing per base. In our study, which used a Pippin Prep (Sage) to size-select a 240 bp insert that was then sequenced with 100 bp reads, there was a ~20 % loss of data during the overlapping stage (Additional file 3). The high read depth required to call rare alleles below 0.5 % frequency makes it economically unfeasible to sequence entire large genomes using PELE-Seq. In addition, the PELE-Seq method is not currently applicable for population genomic studies where minor alleles are believed to provide little useful information.

The PELE-Seq method can detect the majority of rare alleles that are present in a library at a frequency of 0.4 %, but it is very difficult to detect rare SNPs present at or below 0.2 % frequency, even with very high depths of coverage (60,000× OPE), as these SNPs are impossible to distinguish from background PCR errors in the library. For this reason, the ideal number of individuals in a pooled sample would be below 500 in order to maintain rare allele frequencies that are detectable with PELE-Seq.

PELE-Seq is very well-suited for sequencing rare alleles in a small, localized genomic space, such as a gene amplicon. For example, a research project seeking to determine the somatic mutation rate of an organism would be very reasonable economically with PELE-Seq. In this instance, 20,000× coverage of a 1 Kb amplicon could currently be sequenced with 0.062 % of a lane on a 100 bp paired-end Illumina Hiseq 2500 run (1 Kb × 20,000× depth/100 bp reads/400 M reads/lane/0.80 loss during overlap). PELE-Seq is also an ideal method for investigating rare SNPs in the small genomes of mitochondria and viruses, whose genome sizes are in the Kb size range. Similarly, t 16.7 Kb human mitochondrial genome can currently be sequenced to a depth of 20,000× with 1.0 % of a lane on a 100 bp paired-end Illumina Hiseq 2500 run.

Targeted DNA capture libraries are also relatively affordable to sequence with PELE-Seq. A targeted DNA capture library containing 3000 amplicons from a single individual or a population can be sequenced to 20,000× read depth with 18.8 % of a lane on a 100 bp paired-end run on an Illumina Hiseq 2500. However, large-scale eukaryotic genome projects such as eukaryotic exome and transcriptome sequencing would be very expensive to sequence to an ideal read depth for PELE-Seq. To achieve 20,000× OPE read depth of the diploid human exome, it would require 16 lanes on a 250 bp paired-end Illumina Hiseq 2500 run, which is currently very expensive (81 Mb exome × 20,000× read depth/250 bp reads/400 M reads/lane/0.80 loss during overlap). PELE-Seq libraries of genomic regions in the Mb range would need to be sequenced at lower than ideal read depths, based on available funding.

In our study we found that the cost of PELE-Seq data was approximately equal to that of standard DNA-Seq data when the results are based on the amount of rare alleles detected, starting the same raw reads. The PELE-Seq data is completely error-free whereas the standard data contains ~30–50 % errors. We’ve empirically observed that reducing the background noise in a bam file caused by sequencing errors, through the use of overlapping reads, improves the ability of the rare variant-calling program LoFreq to detect rare alleles. This observation is presumably due to the fact that LoFreq calculates information about the amount of background noise in a library, and uses this information to determine whether to call a non-reference allele as an error or a true SNP [16]. The fact that PELE-Seq can identify the same number of rare alleles, with no false-positive SNPs, as the standard methods which generate numerous false positive SNPs, makes PELE-Seq the logical choice for research projects where the main goal of the project is to detect rare alleles, such as with tumor amplicon sequencing and high-throughput screens for rare mutations.

We have used PELE-Seq to identify several rare alleles in a wild *C. remanei* population whose frequencies have increased dramatically as a result of laboratory cultivation. We identified a rare G > C transversion upstream of the promoter of *ugt-5* that was increased in frequency 44× in the lab-adapted strain, compared to the wild strain. UGT enzymes catalyze the addition of a glucuronic acid moiety onto xenobiotics and drugs to enhance their elimination. The UGT pathway is a major pathway responsible for the removal of most drugs, toxins, and foreign substances [26]. The striking increase in the frequency of this rare mutation after lab adaptation suggests that the surrounding genomic region is under positive selection. One possibility is that a change in *ugt-5* expression may confer a growth advantage on the laboratory-grown nematodes by increasing their ability to process and eliminate the bleach ingested during the hatchoff procedures. With PELE-Seq, it is possible to know that the *ugt-5* SNP was present at a very low frequency in the wild population, and is not a *de novo* mutation.

With PELE-Seq we were able to identify91 putative *de novo* mutations in the lab-adapted *C. remanei* population that were completely absent in the wild population at read depths of 900× OPE. Many of these SNPs were present at significant frequencies (5–15 %) in the lab population despite being absent in the wild population. We also identified 19 SNPs in the wild population that were undetectable in the lab-adapted population; these were all present at frequencies below 6 % in the wild population and were presumably lost due to bottlenecking and genetic drift. The different profiles of the SNPs found only in the wild population and those found only in the lab-adapted population provides supporting evidence that the method is detecting actual biological changes in the rare allele frequencies of the populations. If the large increases in frequency seen with the putative *de novo* SNPs were due to some sequencing artifact such as biased PCR amplification, then the allele frequencies of the SNPs present only in the wild and lab-adapted populations would have similar characteristics since they were sequenced exactly the same way. The fact that the putative *de novo* alleles that we’ve detected can increase dramatically in frequency after only 23 generations implies that *de novo* alleles are a valuable source of genetic diversity for populations adapting to change. In this study, we sampled only a very small fraction (~1/500) of the *C. remanei* genome with RAD-Seq, and discovered multiple instances of apparent selection taking place.

## Conclusions

We have demonstrated that the PELE-Seq method of variant calling is highly specific at detecting rare SNPs found at below 1 % in a population. There were zero instances of false positive SNPs called from PELE-sequenced control *E. coli* libraries containing rare alleles present at known frequencies, whereas standard NGS DNA-Seq libraries contained 30–50 % false-positive SNPs. The PELE-Seq method makes it possible to know with 100 % certainty that minor alleles identified by sequencing are actually present in a population and not due to sequencing or PCR error. PELE-Seq can also be used to detect putative *de novo* mutations that are present in one population but absent in another. As a proof of principle, we have used PELE-Seq to identify rare mutations found in lab-adapted strains of *C. remane*i nematode worms. We identified SNPs in the lab-adapted worms that had dramatically increased in frequency after 23 generations in the lab, as well as SNPs present at 5–15 % frequency in the lab-adapted population that were completely absent in the wild population. This research demonstrates that model organisms grown in a laboratory can become genetically distinct from wild populations in a short period of time, and that care must be taken when generalizing from conclusions drawn from research involving lab-reared organisms. These conclusions would not have been possible without PELE-Seq because rare alleles present below 1 % are undetectable with standard DNA-Seq methods, even with high read depths and quality scores.

## Methods

### *Caenorhabditis remanei* population harvesting and cultivation

Isofemale strains originating from 26 wild mating pairs of *C. remanei* worms were expanded to a population size of 2000 following the initial mating. All worms collected, and those in the experiment described below, were grown on nematode growth media (NGM) seeded with *E. coli* strain OP50. All collected strains were frozen within three generations of collection to minimize lab adaptation. To create a cohort representative of naturally segregating variation for experimental evolution, we thawed samples from each of the 26 isofemale strains and crossed them in a controlled fashion to promote equal contributions from all strains, including from mitochondrial genomes and Y chromosomes. The resulting genetically heterogeneous population was frozen after creation and was the ancestral population used for the experiment.

A lab-adaptation strain consisting of 1000–2000 mating individuals was propagated. The control populations were randomly culled to 1000 L1 larvae during each selective generation, for 23 generations. Each population was frozen (N ≥ 100,000 individuals) periodically to retain a record of evolutionary change in the populations and to ensure that worms did not lose the ability to survive freeze and thaw. Approximately 5000 individuals from the frozen populations were thawed to continue the evolution experiment, while the remaining 95,000 worms remained frozen for future phenotyping and genetic and genomic analyses. Populations were thawed for selection after a minimum of 24 h at −80 °C. Freezing occurred a total of 2 times during lab-adaptation selection. The lab-adapted population was also subjected to 11 rounds of bleaching/age-synchronization.

### DNA isolation

*C. remanei* genomic DNA was isolated using the DNeasy Tissue Kit (Qiagen). *E. coli* genomic DNA was acquired from REL606 strain (provided by the Bohannan lab, UO) and from W3110 strain (life technologies).

### *E. coli* spike-in PELE-RAD library construction for Illumina sequencing

Serial dilution of *E. coli* W3110 DNA with *E. coli* Rel606 DNA was performed to generate 4 spike-in libraries with average rare allele frequencies ranging from 1:200 to 1:500, at a concentration of 0.8 ng/μl. The spike-in mixtures were generated as a serial dilution to represent a titration curve of rare allele frequencies. The true allele frequencies of the libraries were determined by sequencing and were found to represent a dilution series of rare allele frequencies, as expected. All dilutions were concentrated with a SpeedVac to 40 μl.

The 6 DNA samples (4 spike-in and 2 pure libraries) were sequenced with 2 barcodes per sample. Each library was generated with 300 ng DNA, representing a copy number of ~60 million individual *E. coli* cells.

Restriction-Site Associated DNA (RAD) Sequencing was used to reduce the complexity of the *C. remanei* genome [17]. RAD-Seq libraries were prepared according to the standard protocol with two modifications:

1. A 100 bp insert size was created by size-selection of a tight 240 bp band. The libraries were then sequenced with 100 bp paired-end reads to generate two completely-overlapping reads. Precise size-selection of the libraries is important because the paired-end reads must completely overlap in order to be merged into a consensus sequence. Any bases overhanging the overlapped sequence are excluded from the analysis.
2. During the amplification step, each PCR reaction contained a mixture of two independently-barcoded adapters. After DNA amplification, the resulting libraries contained a mixture of two separately-barcoded adapters on the same end of the DNA molecules.

For this application we used the restriction enzyme SbfI, which cuts the sequence CCTGCAGG. The SbfI site occurs ~70 times in the *E. coli* genome, leading to the creation of 140, 100 bp RAD tags, spanning 14 Kb of total DNA.

DNA from each dilution was digested for 60 min at 37C in a 50 μL reaction volume containing 5.0 μL Buffer 4, 10 units (U) *SbfI-*HF (New England Biolabs [NEB]). Samples were heat-inactivated for 20 min at 65 C. 2.0 μL of barcoded *SbfI*-P1 adapter mixture (100 n*M*), a modified Illumina© adapter (2006 Illumina, Inc., all rights reserved; top oligo: 5′-Phos-AATGATACGGCGACCACCGAGATCTACACTCTTTCCCTACACGACGCTCTTCCGATCTxxxxxxTGC*A 3′[xxxxxx = barcode (mixture of two barcodes per sample), * = phosphoro-thioate bond]; bottom oligo: 5′-Phos-xxxxxxAGATCGGAAGAGCGTCGTGTAGGGAAAGAGTGTAGATCTCGGTGGTCGCCGTATCAT*T), was added to each sample along with 0.6 ml rATP (100 mM, Promega), 1.0 μl 10× NEB Buffer 4, 0.5 μl (1000 U) T4 DNA Ligase (high concentration, NEB), 3.9 μl H_2_O and incubated at room temperature (RT) for 30 min. Samples were again heat-inactivated for 20 min at 65C, combined, and randomly sheared (Bioruptor) to an average size of 140 bp. The sheared sample was purified using Agencourt AMPure XP beads at a 1× volume. The Quick Blunting Kit (NEB) was used to blunt the ends of the DNA in a 50 μl reaction volume, and the sample was purified using Agencourt AMPure XP beads at a 1× volume. The sample was incubated at 37C for 30 min with 10 U Klenow Fragment (3′–5′ exo-, NEB) in a 50 μl reaction volume with 5.0 μl NEB Buffer 2 and 1.0 μl dATP (10 mM, Fermentas), to add 3’ adenine overhangs to the DNA. After another 1× bead purification, 1.0 ml of Paired-End-P2 Adapter (PE-P2; 10 mM), a divergent modified Illumina© adapter (2006 Illumina, Inc., all rights reserved; top oligo: 5′-Phos-GATCGGAAGAGCGGTTCAGCAGGAATGCCGAGACCGATCAGAACAA-3′, bottom oligo: 5′-CAAGCAGAAGACGGCATACGAGATCGGTCTCGGCATTCCTGCTGAACCGCTCTTCCGATC*T-3′), was ligated to the DNA fragments at RT. The sample was purified and eluted in 40 μl elution buffer. Ten separate PCR amplifications were performed with each of the 6 samples, each using 4 μl of eluate as template, in a 50 μl volume with 25 μl Phusion Master Mix (NEB) and 1.0 μl modified Illumina© amplification primer mix (10 mM, 2006 Illumina, Inc., all rights reserved; P1-forward primer: 5′ AATGATACGGCGACCACCGAGATCTACACTCTTTCCCTACACGACGCTCTTCCGATC*T 3′, P2-reverse primer: 5′ CAAGCAGAAGACGGCATACG*A 3′). Phusion PCR settings followed product guidelines (NEB) for a total of 18 cycles with an annealing temperature of 65C. The 6 libraries were then pooled, cleaned through a QIAquick Spin column (Qiagen), and size selected with a Pippin Prep (Sage), collecting a tight band of DNA of 240 bp size. The sample was diluted to 1 nM and sequenced on the Paired-end module of an Illumina HiSeq 2500 following Illumina protocols for 100 bp reads.

### *C. remanei* PELE-RAD library construction for Illumina sequencing

Restriction-Site Associated DNA (RAD) Sequencing was used to reduce the complexity of the *C. remanei* genome [17] RAD-Seq libraries were prepared according to the standard protocol with two modifications:

1. A 100 bp insert size was created by size-selection of a tight 240 bp band. The libraries were then sequenced with 100 bp paired-end reads to generate two completely-overlapping reads. Precise size-selection of the libraries is important because the paired-end reads must completely overlap in order to be merged into a consensus sequence. Any bases overhanging the overlapped sequence are excluded from the analysis.
2. During the amplification step, each PCR reaction contained a mixture of two independently-barcoded adapters. After DNA amplification, the resulting libraries contained a mixture of two separately-barcoded adapters on the same end of the DNA molecules.

For this application we used the restriction enzyme PacI, which has an AT-rich cut site. The complexity of the PacI RAD library was further reduced by digestion with NlaIII, which destroyed ~30 % of the total RAD tags. The PacI cut site AATTAATT occurs 2044 times in the *C. remanei* genome, leading to the creation of 4088, 100 bp RAD tags, spanning 409 Kb of total DNA. After NlaII digestion, 287 Kb of DNA sequence remained and was sequenced with RAD-Seq at 800× PE coverage. RAD tags were present at approximately every 10 kb throughout the genome.

For the *C. remanei* study, there were 2 samples (“wild” and “lab-adapted”) sequenced with two barcodes each. The ancestral population was produced from 39 isofemale strains, and each sample contained ~1000 individual worms.

Genomic DNA (2.0 μg) from each population was digested for 60 min at 37C in a 50 μL reaction volume containing 5.0 μL Buffer 1, 10 units (U) *PacI* (New England Biolabs [NEB]), and 0.5 μl 100× BSA (NEB). Samples were heat-inactivated for 20 min at 65 C. 1.0 μL of barcoded *PacI*-P1 adapter mixture (100 n*M*), a modified Illumina© adapter (2006 Illumina, Inc., all rights reserved; top oligo: 5′-ACACTCTTTCCCTACACGACGCTCTTCCGATCTxxxxx(xx)A*T -3′[xxxxx(xx) = barcode (TACGT, AGATCGA - ancestor; CTGCAA, GCTAGTC –evolved control, mixture of two barcodes per sample), * = phosphoro-thioate bond]; bottom oligo: 5′-Phos-xxxxx(xx)AGATCGGAAGAGCGTCGTGTAGGGAAAGAGTG*T), was added to each sample along with 0.6 ml rATP (100 mM, Promega), 1.0 μl 10× NEB Buffer 4, 0.5 μl (1000 U) T4 DNA Ligase (high concentration, NEB), 3.9 μl H_2_O and incubated at room temperature (RT) for 30 min. Samples were again heat-inactivated for 20 min at 65C, combined, and randomly sheared (Bioruptor) to an average size of 140 bp. The sheared sample was purified using a QIAquick Spin column (Qiagen) and run out on a 1.25 % agarose (Sigma), 0.5× TBE gel. A tight band of DNA from 130–150 bp was isolated with a clean razor blade and purified using the MinElute Gel Extraction Kit (Qiagen). The Quick Blunting Kit (NEB) was used to blunt the ends of the DNA in a 25 μl reaction volume containing 2.5 μl 10× Blunting Buffer, 2.5 μl dNTP Mix and 1.0 μl Blunt Enzyme Mix. The sample was purified and incubated at 37C for 30 min with 10 U Klenow Fragment (3′–5′ exo-, NEB) in a 50 μl reaction volume with 5.0 μl NEB Buffer 2 and 1.0 μl dATP (10 mM, Fermentas), to add 3’ adenine overhangs to the DNA. After another purification, 1.0 ml of Paired-End-P2 Adapter (PE-P2; 10 mM), a divergent modified Illumina© adapter (2006 Illumina, Inc., all rights reserved; top oligo: 5′-Phos-GATCGGAAGAGCGGTTCAGCAGGAATGCCGAGACCGATCAGAACAA-3′, bottom oligo: 5′-CAAGCAGAAGACGGCATACGAGATCGGTCTCGGCATTCCTGCTGAACCGCTCTTCCGATC*T-3′), was ligated to the DNA fragments at RT. The sample was purified and eluted in 50 μl. The eluate was digested again with NlaIII to reduce library complexity. The sample was column-purified and eluted in 10 μl elution buffer. Two separate PCR amplifications were performed with each sample, each using 5 μl of eluate as template, in a 50 μl volume with 25 μl Phusion Master Mix (NEB) and 1.0 μl modified Illumina© amplification primer mix (10 mM, 2006 Illumina, Inc., all rights reserved; P1-forward primer: 5′ AATGATACGGCGACCACCGAGATCTACACTCTTTCCCTACACGACGCTCTTCCGATC*T 3′, P2-reverse primer: 5′ CAAGCAGAAGACGGCATACG*A 3′). Phusion PCR settings followed product guidelines (NEB) for a total of 17 cycles with an annealing temperature of 65C. The wild and lab-adapted DNA libraries were pooled and cleaned through a column and gel purified, excising a tight band of DNA of 240 bp size. The sample was diluted to 1 nM and sequenced on the Paired-end module of the Genome Analyzer II following Illumina protocols for 100 bp reads.

### Data analysis of standard paired-end data

Raw reads were cleaned with Stacks process_radtags (v0.99993) to remove low quality bases present at the ends of reads (http://catchenlab.life.illinois.edu/stacks/). Reads were aligned to reference genomes with Bowtie (v2.2.1). Sam files were converted to bams and mpileups with Samtools (v0.1.18). Bam files were sorted with Picard SortSam (v1.115). Base quality score recalibration (BQSR) was performed using GATK (v2.6-4). Low-frequency variants were called with LoFreq (v2.0.0-rc-1) using default mode [16], with a minimum allele frequency cutoff of AF = 0.002.

### Data analysis of PELE-Seq data

An overview of the recommended PELE-Seq analysis workflow and optimized variant-calling parameters are provided in Additional file 6. Basic scripts are written as a shell pipeline, and are included in Additional file 8.

Raw reads were cleaned with Stacks process_radtags (v0.99993) to remove low quality bases present at the ends of reads. (http://catchenlab.life.illinois.edu/stacks/). Overlapping paired-end reads were merged with SeqPrep (v0.1) (https://github.com/jstjohn/SeqPrep). Overhanging reads were trimmed from merged reads with BBMap (v32.07) (http://sourceforge.net/projects/bbmap/), using a quality score cutoff of Q60. Reads were aligned to reference genomes with Bowtie (v2.2.1) (http://bowtie-bio.sourceforge.net/index.shtml). Sam files were converted to bams and mpileups with Samtools (v0.1.18) (http://www.htslib.org/). Bam files were sorted with Picard SortSam (v1.115) (http://broadinstitute.github.io/picard/).

PELE-Seq SNPs were called using a multi-step variant calling approach to incorporate information from the barcoding and overlapping steps, without a large drop in sensitivity. The PELE-Seq analysis pipeline is based on creating two lists of SNP calls for each library: List A contains SNPs called from the merged overlapping reads (ORP data) and List B contains SNPs that were called separately in both individually-barcoded library called using less stringent parameters during SNP-calling, also with overlapping reads.

To create List A, SNPs were called from overlapped paired-end read data using the program Lofreq (v2.1.2) (http://csb5.github.io/lofreq/) with a minimum allele frequency cutoff of AF = 0.0055-0.002 (see Additional file 3). The two separately barcoded files were pooled prior to variant calling using Picard MergeSamFiles (v1.115).

To create List B, SNPs were called from each barcoded sample separately using LoFreq at a decreased stringency mode (-J –B options) with a minimum allele frequency cutoff of 0.0005-0.002 and Q Score cutoff of 150–820 (see Additional file 3). SNPs present in both barcode files were recorded in “List B”.

To generate the final list of PELE-Seq SNPs, List A and B were added.

In general, the allele frequency cutoff of 0.002 was found to always eliminate false positive mutations in the data, as did using a Q score cutoff of 700 for the separately-barcoded files, when read depths above 1000× OPE per barcode were used. If the background PCR error rate of the ORP data is unknown, one option for achieving very high-quality SNP calls is to call SNPs only if they are present in both Lists A and B, however this decreases the sensitivity of the method.

### Detection of putative “*de novo*” alleles

Raw reads were cleaned with Stacks process_radtags (v0.99993) to remove low quality bases present at the ends of reads (http://catchenlab.life.illinois.edu/stacks/). Overlapping paired-end reads were merged with SeqPrep (v0.1) (https://github.com/jstjohn/SeqPrep). Overhanging reads were trimmed from merged reads with BBMap (v32.07) (http://sourceforge.net/projects/bbmap/). Reads were aligned to reference genomes with Bowtie (v2.2.1) (http://bowtie-bio.sourceforge.net/index.shtml). Sam files were converted to bams and mpileups with Samtools (v0.1.18) (http://www.htslib.org/). Bam files were sorted with Picard SortSam (v1.115) (http://broadinstitute.github.io/picard/).

Rare alleles present in one sample and absent in another were called using Seurat Somatic (v2.5) [21] Alleles were called with a minimum read depth of 900 OPE and were filtered to remove any allele that appeared in the original population with an allele frequency above 0.000.

### Visualization of sequencing data

Sequencing data is visualized using circos [27], the Integrative Genomics Viewer (IGV) [28, 29], and the R packages Sushi [30] and ggplot2 [31].

## Additional files

Additional file 1: The 64 control alleles present in the control *E. coli* “spike-in” libraries. The rare SNPs present in the control libraries were determined by sequencing the pure *E. coli* K12 substrain W3110 and aligning it to the *E. coli* B substrain Rel606 genome. (PDF 15 kb) Additional file 2: Sequencing reads from the pure W3110 and Rel606 *E. coli* substrains. The purity of the original DNA samples was verified through sequencing by aligning the sequencing reads from both *E. coli* substrains to the Rel606 genome. The “true positive” SNPs were found to be present at 100 % frequency in the W3110 DNA, and all other positions contained reference bases at 100 % frequency. (PDF 604 kb) Additional file 3: SNP-calling results from *E. coli* spike-in libraries using the PELE-Seq, ORP, and Standard DNA-Seq methods. The performance of the PELE-Seq, ORP, and standard DNA-Seq methods at detecting rare alleles at various read depths. SNP results for spike-in libraries 1–4 are reported, as well as read depths and other metrics for the libraries. Various allele frequency and quality score cutoffs were tested to optimize rare variant identification with the method, and the SNP-calling results are reported here. Recommended workflow and SNP-calling parameters are reported here and in Additional file 6. (XLSX 54 kb) Additional file 4: Control SNPs detected with PELE-Seq, standard DNA-Seq, and the ORP method. Rare alleles present at 0.42 % frequency in a control *E. coli* spike-in library were sequenced with PELE-Seq, the ORP method, and the standard DNA-Seq method at 10,000× OPE depth of coverage per barcode (24,000× depth per barcode of non-overlapped reads). PELE-Seq and the ORP method are 100 % specific and the standard DNA-Seq method is 75 % specific. This data is also included as a spreadsheet in Additional file 3. (PDF 1109 kb) Additional file 5: PCR errors that result in false positive SNPs when using unfiltered ORP data. Overlapping paired-end read libraries sequenced to 10,000× OPE depth contained 109 false positive SNPs when rare variants were called with Lofreq, using default parameters with no minimum allele frequency cutoff. These putative PCR errors are predominately C > T transitions are are found in distinct clusters throughout the genome. This data is also included as a spreadsheet in Additional file 3. (PDF 18 kb) Additional file 6: Recommended PELE-Seq workflow and SNP-calling parameters. An overview of the PELE-Seq analysis pipeline is outlined and optimized variant-calling parameters are provided for various read depths. (XLSX 8 kb) Additional file 7: SNP results from PELE-sequencing of wild and lab-adapted *C. remanei* populations. Rare SNPs in the wild and lab-adapted C. remanei populations were detected with PELE-Seq at 2000× OPE read depth. Alleles present in both populations are listed, as well as those found in either the wild or lab-adapted population alone. Read pileups are listed for SNPs are absent in one population (putative *de novo* SNPs and SNPs lost during lab-adaptation). (XLSX 722 kb) Additional file 8: Basic PELE-seq SNP-calling scripts. The basic commands used for PELE-Seq analysis are provided as a shell script. (TXT 11 kb)

## Abbreviations

AF: allele frequency
ddRAD: double-digest RAD
GBS: genotype by sequencing
NGS: next-generation sequencing
OPE: overlapping paired-end
ORPs: overlapping read pairs
PELE-Seq: paired-end low error sequencing
RAD-Seq: restriction-site associated DNA
SNPs: single nucleotide polymorphisms
TILLING: targeting induced local lesions in genomes
