# Supplementary material for: High-specificity detection of rare alleles with Paired-End Low Error Sequencing (PELE-Seq)
Source: BMC Genomics. 2016 Jun 14;17:464. doi: 10.1186/s12864-016-2669-3 (PMC4908710; doi:10.1186/s12864-016-2669-3)
Supplement: Additional file 1: — The 64 control alleles present in the control E. coli “spike-in” libraries. The rare SNPs present in the control libraries were determined by sequencing the pure E. coli K12 substrain W3110 and aligning it to the E. coli B substrain Rel606 genome. (PDF 15 kb) [file 12864_2016_2669_MOESM1_ESM.pdf]

ID	Position		Ref	Alt
1	94900	.	T	A
2	94966	.	T	C
3	175590	.	T	C
4	175596	.	A	G
5	175737	.	C	T
6	221029	.	C	T
7	536957	.	C	T
8	561460	.	T	C
9	741104	.	T	C
10	816895	.	G	A
11	817018	.	A	G
12	853386	.	C	A
13	853407	.	G	A
14	853410	.	C	T
15	853521	.	T	C
16	1007276	.	T	C
17	1083049	.	T	C
18	1083052	.	C	T
19	1083053	.	A	G
20	1083055	.	C	T
21	1083076	.	A	G
22	1171113	.	C	T
23	1171239	.	C	T
24	2146885	.	A	G
25	2146888	.	C	G
26	2146891	.	A	G
27	2162714	.	A	C
28	2167199	.	G	C
29	2167359	.	A	G
30	2440038	.	A	G
31	2440136	.	G	A
32	2468768	.	A	G
33	2468789	.	T	C
34	2468858	.	T	C
35	2468873	.	T	C
36	2468900	.	A	G
37	2728328	.	C	T
38	2728331	.	A	T
39	2728367	.	A	C
40	2728442	.	C	T
41	2728454	.	C	G
42	2728482	.	G	A
43	2920577	.	A	G
44	2920601	.	G	A

45	2920616	.	A	G
46	2920646	.	A	G
47	2920697	.	G	A
48	3269492	.	A	G
49	3269621	.	C	T
50	4010517	.	C	T
51	4010523	.	A	G
52	4010538	.	C	T
53	4010580	.	C	T
54	4354970	.	T	G
55	4399889	.	G	A
56	4399934	.	C	A
57	4399937	.	A	G
58	4399940	.	A	G
59	4399970	.	G	A
60	4399976	.	T	G
61	4399979	.	C	A
62	4458342	.	C	T
63	4458362	.	G	A
64	4458477	.	C	T
