# Supplementary material for: High-specificity detection of rare alleles with Paired-End Low Error Sequencing (PELE-Seq)
Source: BMC Genomics. 2016 Jun 14;17:464. doi: 10.1186/s12864-016-2669-3 (PMC4908710; doi:10.1186/s12864-016-2669-3)
Supplement: Additional file 2: — Sequencing reads from the pure W3110 and Rel606 E. coli substrains. The purity of the original DNA samples was verified through sequencing by aligning the sequencing reads from both E. coli substrains to the Rel606 genome. The “true positive” SNPs were found to be present at 100 % frequency in the W3110 DNA, and all other positions contained reference bases at 100 % frequency. (PDF 604 kb) [file 12864_2016_2669_MOESM2_ESM.pdf]

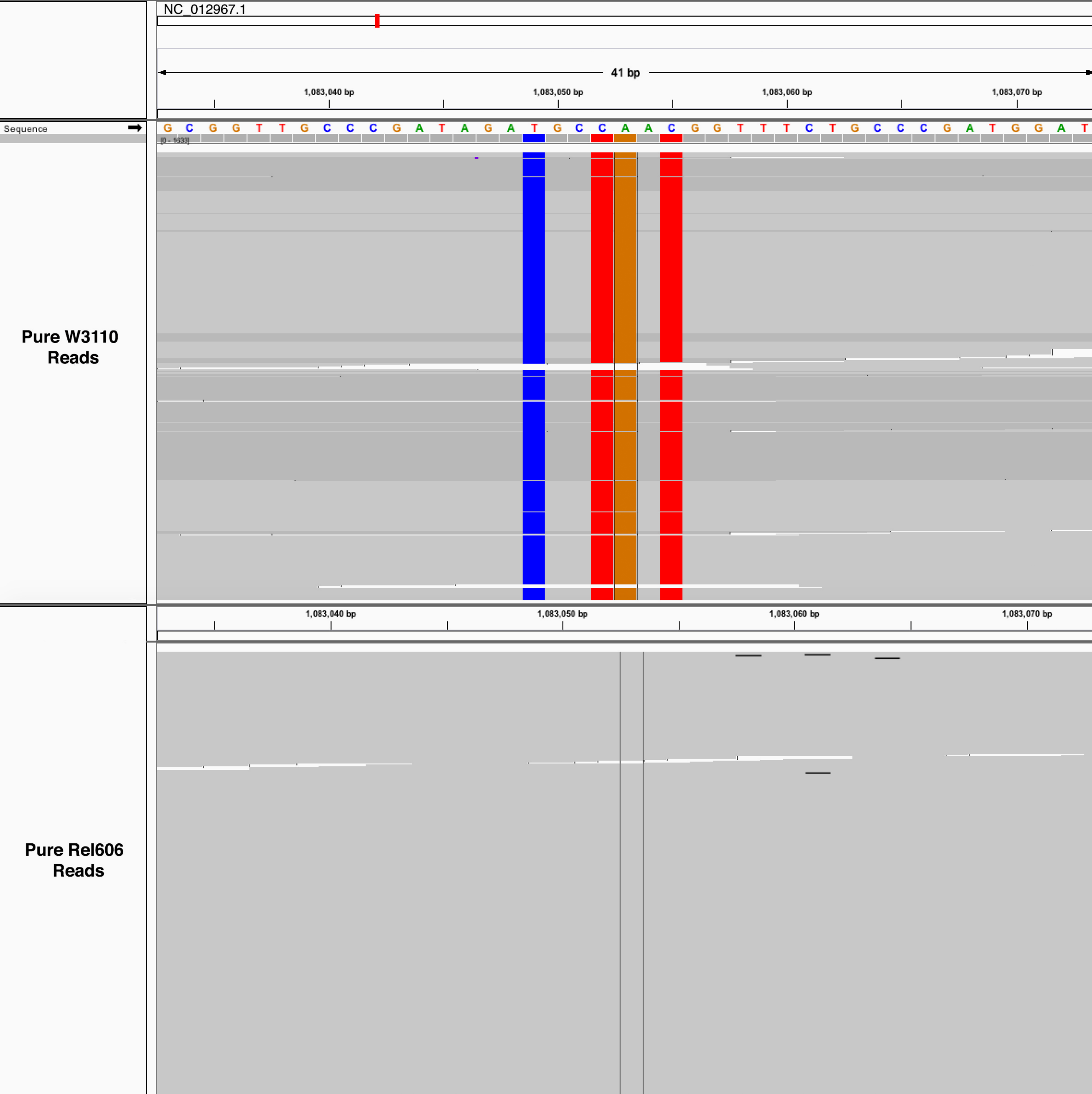
NC_012967.1
41 bp
1,083,040 bp
1,083,050 bp
1,083,060 bp
1,083,070 bp
Sequence
G C G G T T G C C C G A T A G A T G C C A A C G G T T T C T G C C C G A T G G A T
[0 - 1633]
Pure W3110 Reads
1,083,040 bp
1,083,050 bp
1,083,060 bp
1,083,070 bp
Pure Rel606 Reads
