# Supplementary material for: High-specificity detection of rare alleles with Paired-End Low Error Sequencing (PELE-Seq)
Source: BMC Genomics. 2016 Jun 14;17:464. doi: 10.1186/s12864-016-2669-3 (PMC4908710; doi:10.1186/s12864-016-2669-3)
Supplement: Additional file 4: — Control SNPs detected with PELE-Seq, standard DNA-Seq, and the ORP method. Rare alleles present at 0.42 % frequency in a control E. coli spike-in library were sequenced with PELE-Seq, the ORP method, and the standard DNA-Seq method at 10,000× OPE depth of coverage per barcode (24,000× depth per barcode of non-overlapped reads). PELE-Seq and the ORP method are 100 % specific and the standard DNA-Seq method is 75 % specific. This data is also included as a spreadsheet in Additional file 3. (PDF 1109 kb) [file 12864_2016_2669_MOESM4_ESM.pdf]

PELE-Seq Method

True Positives

ID	Position	Ref	Alt
1	94900	T	A
2	94966	T	C
3	175590	T	C
4	175596	A	G
5	175737	C	T
6	221029	C	T
7	741104	T	C
8	817018	A	G
9	853386	C	A
10	853407	G	A
11	853410	C	T
12	1007276	T	C
13	1083052	C	T
14	1083053	A	G
15	1083055	C	T
16	1171239	C	T
17	2146885	A	G
18	2146888	C	G
19	2146891	A	G
20	2162714	A	C
21	2167199	G	C
22	2440038	A	G
23	2440136	G	A
24	2468768	A	G
25	2468789	T	C
26	2468858	T	C
27	2728328	C	T
28	2728331	A	T
29	2728367	A	C
30	2728482	G	A
31	2920697	G	A
32	3269492	A	G
33	3269621	C	T
34	4010523	A	G
35	4010538	C	T
36	4010580	C	T
37	4399889	G	A
38	4399970	G	A
39	4399979	C	A
40	4458342	C	T
41	4458362	G	A
42	4458477	C	T

No False Positives

ORP Method

True Positives

ID	Position	Ref	Alt
1	94900	T	A
2	94966	T	C
3	175590	T	C
4	175596	A	G
5	221029	C	T
6	741104	T	C
7	817018	A	G
8	853386	C	A
9	853407	G	A
10	853410	C	T
11	1083052	C	T
12	1083053	A	G
13	1083055	C	T
14	1171239	C	T
15	2146885	A	G
16	2146888	C	G
17	2146891	A	G
18	2162714	A	C
19	2440038	A	G
20	2440136	G	A
21	2468768	A	G
22	2468789	T	C
23	2468858	T	C
24	2728328	C	T
25	2728331	A	T
26	2728367	A	C
27	2728482	G	A
28	2920697	G	A
29	3269492	A	G
30	4010523	A	G
31	4010538	C	T
32	4010580	C	T
33	4399889	G	A
34	4399970	G	A
35	4458342	C	T
36	4458362	G	A
37	4458477	C	T

No False Positives

Standard DNA-Seq, Q30

True Positives

ID	Position	Ref	Alt
1	94900	T	A
2	94966	T	C
3	175590	T	C
4	175596	A	G
5	175737	C	T
6	561460	T	C
7	741104	T	C
8	817018	A	G
9	853386	C	A
10	853410	C	T
11	1083049	T	C
12	1083052	C	T
13	1083053	A	G
14	1083055	C	T
15	1083076	A	G
16	1171239	C	T
17	2146891	A	G
18	2162714	A	C
19	2167199	G	C
20	2440136	G	A
21	2468768	A	G
22	2468789	T	C
23	2468873	T	C
24	2468900	A	G
25	2728328	C	T
26	2728331	A	T
27	2728367	A	C
28	2920697	G	A
29	3269492	A	G
30	3269621	C	T
31	4010538	C	T
32	4010580	C	T
33	4399889	G	A
34	4458342	C	T
35	4458362	G	A
36	4458477	C	T

False Positives

ID	Position	Ref	Alt
1	175542	G	A
2	221119	G	A
3	817075	G	A
4	2146819	T	C
5	2146924	G	A
6	2147025	C	A
7	2920673	A	G
8	2920679	G	C
9	2920763	A	G
10	3016457	C	T
11	3402330	G	T
12	4458403	A	G
