# Supplementary material for: High-specificity detection of rare alleles with Paired-End Low Error Sequencing (PELE-Seq)
Source: BMC Genomics. 2016 Jun 14;17:464. doi: 10.1186/s12864-016-2669-3 (PMC4908710; doi:10.1186/s12864-016-2669-3)
Supplement: Additional file 5: — PCR errors that result in false positive SNPs when using unfiltered ORP data. Overlapping paired-end read libraries sequenced to 10,000× OPE depth contained 109 false positive SNPs when rare variants were called with Lofreq, using default parameters with no minimum allele frequency cutoff. These putative PCR errors are predominately C > T transitions are are found in distinct clusters throughout the genome. This data is also included as a spreadsheet in Additional file 3. (PDF 18 kb) [file 12864_2016_2669_MOESM5_ESM.pdf]

ID	Position	Ref	Alt	Allele Freq	Read Depth
1	94863	G	T	0.0010	19591
2	94894	C	T	0.0010	16127
3	175718	G	A	0.0010	28271
4	221034	C	T	0.0012	22815
5	221159	C	T	0.0009	46592
6	221160	G	A	0.0007	46441
7	221165	C	T	0.0005	45214
8	221172	C	T	0.0007	43794
9	458683	C	T	0.0011	38880
10	458684	C	T	0.0009	38857
11	458691	C	T	0.0007	37298
12	458692	G	T	0.0005	37143
13	458700	C	T	0.0018	36968
14	458702	C	T	0.0010	36778
15	458710	A	T	0.0007	34167
16	536836	C	T	0.0007	32560
17	536958	C	A	0.0010	23712
18	585385	G	A	0.0011	39161
19	760272	C	T	0.0008	34606
20	760286	G	A	0.0005	37360
21	760432	G	A	0.0016	21793
22	816919	C	T	0.0009	20251
23	817014	C	T	0.0010	30495
24	817016	G	A	0.0009	30625
25	817021	G	A	0.0009	32926
26	817031	G	A	0.0009	34027
27	817032	G	A	0.0008	34129
28	822923	C	T	0.0011	25571
29	823011	C	T	0.0010	30064
30	823027	G	A	0.0009	27602
31	898828	G	T	0.0008	21257
32	979601	C	T	0.0008	47640
33	1007316	G	A	0.0010	45634
34	1007325	G	A	0.0007	40041
35	1082964	C	T	0.0009	28841
36	1171244	G	A	0.0010	22093
37	1678037	G	C	0.0013	40578
38	1678136	G	A	0.0009	39472
39	1809726	C	T	0.0008	36286
40	1809728	C	T	0.0008	36205
41	1809733	G	T	0.0007	36235
42	1884742	G	A	0.0013	32066
43	1910845	C	T	0.0009	15846
44	1910856	G	A	0.0015	15737

45	1910922	A	G	0.0010	32906
46	2032991	A	T	0.0006	53680
47	2033003	G	A	0.0005	53467
48	2033004	C	T	0.0005	53322
49	2106114	G	A	0.0010	26577
50	2106120	G	A	0.0011	25970
51	2106122	T	C	0.0008	25758
52	2162719	C	T	0.0008	21391
53	2162834	G	A	0.0010	27212
54	2162862	G	T	0.0010	23325
55	2194832	A	G	0.0010	33367
56	2194838	C	T	0.0008	34050
57	2194839	G	A	0.0007	34101
58	2194840	C	T	0.0007	34322
59	2194841	G	A	0.0006	34984
60	2194847	A	G	0.0006	37329
61	2194935	G	A	0.0016	34598
62	2194973	C	T	0.0009	38337
63	2194975	T	C	0.0010	37732
64	2194984	G	A	0.0010	34526
65	2379508	G	A	0.0009	26595
66	2410746	G	A	0.0007	17928
67	2410893	G	A	0.0009	38467
68	2440048	G	A	0.0011	26377
69	2468881	G	A	0.0012	28817
70	2468889	T	C	0.0007	29619
71	2522296	G	A	0.0012	23300
72	2859711	G	A	0.0008	42002
73	2932352	T	A	0.0008	27309
74	2932358	C	T	0.0009	26228
75	3002223	C	T	0.0009	15359
76	3008561	T	A	0.0007	27584
77	3008569	G	A	0.0010	26813
78	3016623	G	A	0.0008	25030
79	3041266	C	T	0.0009	32814
80	3041379	G	A	0.0010	27111
81	3061377	C	T	0.0009	31895
82	3119758	C	T	0.0007	67265
83	3119896	G	A	0.0005	48870
84	3246734	T	C	0.0010	26076
85	3246756	C	A	0.0010	25519
86	3402299	G	A	0.0015	47672
87	4010607	G	A	0.0009	27042
88	4010617	G	A	0.0008	27790
89	4010623	G	A	0.0015	28096
90	4010651	G	A	0.0020	24691

91	4164661	G	T	0.0011	35447
92	4164757	G	A	0.0009	51424
93	4164780	G	A	0.0007	53209
94	4164787	G	A	0.0012	49875
95	4284730	C	T	0.0007	31306
96	4284734	C	T	0.0006	31558
97	4313130	C	T	0.0007	31260
98	4355080	G	A	0.0011	33900
99	4355082	T	C	0.0009	33761
100	4355083	G	A	0.0011	33600
101	4399857	C	T	0.0006	37349
102	4399860	G	A	0.0006	37345
103	4458320	C	T	0.0010	42571
104	4458327	C	T	0.0006	46980
105	4458332	C	T	0.0006	48560
106	4458448	C	T	0.0008	38240
107	4481183	T	A	0.0006	43851
108	4481185	C	T	0.0010	44298
109	4481188	G	A	0.0006	45417
